# Development and Validation of a Serologic Test Panel for Detection of Powassan Virus Infection in U.S. Patients Residing in Regions Where Lyme Disease Is Endemic

**DOI:** 10.1128/mSphere.00467-17

**Published:** 2018-01-10

**Authors:** Angela M. Thomm, Anna M. Schotthoefer, Alan P. Dupuis, Laura D. Kramer, Holly M. Frost, Thomas R. Fritsche, Yvette A. Harrington, Konstance K. Knox, Sue C. Kehl

**Affiliations:** a Coppe Laboratories, Waukesha, Wisconsin, USA; b Marshfield Clinic Research Foundation, Marshfield, Wisconsin, USA; c Wadsworth Center, New York State Department of Health, Slingerlands, New York, USA; d Marshfield Clinic, Marshfield, Wisconsin, USA; e Medical College of Wisconsin, Milwaukee, Wisconsin, USA; Icahn School of Medicine at Mount Sinai

**Keywords:** Powassan virus, deer tick virus, enzyme immunoassay, flavivirus, immunofluorescence, serology, tick-borne disease, tick-borne encephalitis

## Abstract

Powassan virus (POWV) is an emerging tick-borne arbovirus presenting a public health threat in North America. POWV lineage II, also known as deer tick virus, is the strain of the virus most frequently found in *Ixodes scapularis* ticks and is implicated in most cases of POWV encephalitis in the United States. Currently, no commercial tests are available to detect POWV exposure in tick-borne disease (TBD) patients. We describe here the development and analytical validation of a serologic test panel to detect POWV infections. The panel uses an indirect enzyme immunoassay (EIA) to screen. EIA-positive samples reflex to a laboratory-developed, POWV-specific immunofluorescence assay (IFA). The analytical sensitivity of the test panel was 89%, and the limit of detection was a plaque reduction neutralization test (PRNT) titer of 1:20. The analytical specificity was 100% for the IgM assay and 65% for the IgG assay when heterologous-flavivirus-positive samples were tested. On samples collected from regions where Lyme disease is endemic, seroprevalence for POWV in TBD samples was 9.4% (10 of 106) versus 2% when tested with non-TBD samples (2 of 100, *P* = 0.034). No evidence of POWV infection was seen in samples collected from a region where Lyme disease was not endemic (0 of 22). This test panel provides a sensitive and specific platform for detecting a serologic response to POWV early in the course of infection when neutralizing antibodies may not be detectable. Combined with clinical history, the panel is an effective tool for identifying acute POWV infection.

**IMPORTANCE** Approximately 100 cases of POWV disease were reported in the United States over the past 10 years. Most cases have occurred in the Northeast (52) and Great Lakes (45) regions (https://www.cdc.gov/powassan/statistics.html). The prevalence of POWV in ticks and mammals is increasing, and POWV poses an increasing threat in a greater geographical range. In areas of the Northeast and Midwest where Lyme disease is endemic, POWV testing is recommended for patients with a recent tick bite, patients with Lyme disease who have been treated with antibiotics, or patients with a tick exposure who have tested negative for Lyme disease or other tick-borne illnesses and have persistent symptoms consistent with posttreatment Lyme disease. Testing could also benefit patients with tick exposure and unexplained neurologic symptoms and chronic fatigue syndrome (CFS) patients with known tick exposure. Until now, diagnostic testing for Powassan virus has not been commercially available and has been limited to patients presenting with severe, neurologic complications. The lack of routine testing for Powassan virus in patients with suspected tick-borne disease means that little information is available regarding the overall prevalence of the virus and the full spectrum of clinical symptoms associated with infection. As *Ixodes scapularis* is the tick vector for Powassan virus and multiple other tick-borne pathogens, including the Lyme disease bacterium, *Borrelia burgdorferi*, the clinical presentations and long-term outcomes of Powassan virus infection and concurrent infection with other tick-borne disease pathogens remain unknown.

## Abstract (precis)

Approximately 100 cases of POWV disease were reported in the United States over the past 10 years. Most cases have occurred in the Northeast (52) and Great Lakes (45) regions (https://www.cdc.gov/powassan/statistics.html). The prevalence of POWV in ticks and mammals is increasing, and POWV poses an increasing threat in a greater geographical range. In areas of the Northeast and Midwest where Lyme disease is endemic, POWV testing is recommended for patients with a recent tick bite, patients with Lyme disease who have been treated with antibiotics, or patients with a tick exposure who have tested negative for Lyme disease or other tick-borne illnesses and have persistent symptoms consistent with posttreatment Lyme disease. Testing could also benefit patients with tick exposure and unexplained neurologic symptoms and chronic fatigue syndrome (CFS) patients with known tick exposure. Until now, diagnostic testing for Powassan virus has not been commercially available and has been limited to patients presenting with severe, neurologic complications. The lack of routine testing for Powassan virus in patients with suspected tick-borne disease means that little information is available regarding the overall prevalence of the virus and the full spectrum of clinical symptoms associated with infection. As *Ixodes scapularis* is the tick vector for Powassan virus and multiple other tick-borne pathogens, including the Lyme disease bacterium, *Borrelia burgdorferi*, the clinical presentations and long-term outcomes of Powassan virus infection and concurrent infection with other tick-borne disease pathogens remain unknown.

## INTRODUCTION

Powassan virus (POWV) is the only North American member of the tick-borne encephalitis complex (TBE-C) of viruses, which are transmitted by the bite of an infected tick. Other members of the TBE-C include the following flaviviruses: tick-borne encephalitis virus (TBEV) in Eastern Europe and Western Asia, Omsk hemorrhagic fever virus in Siberia, Kyasanur Forest disease virus in India, Alkhurma virus in Saudi Arabia, and Louping ill virus in the United Kingdom. TBE-C viruses can cause a wide range of disease in humans, from mild febrile illness with biphasic fever to encephalitis or hemorrhagic fever (1). POWV is composed of two genetically and ecologically distinct lineages (2). Prototype POWV (lineage I) is transmitted primarily by *Ixodes cookei*, a tick with a narrow vertebrate host range that rarely feeds on humans. Powassan virus lineage II, also known as deer tick virus (DTV), is transmitted by *Ixodes scapularis*, a tick with a broad host range that also transmits the infectious agents that cause Lyme disease, anaplasmosis, ehrlichiosis, and babesiosis (3). Since the late 1990s, POWV infections have been reported in the Northeastern and Midwestern parts of the United States as well as in Canada, and incidence is increasing (4). Because the territory of *I. scapularis* is expanding and the prevalence of POWV in ticks and mammals is increasing, POWV poses an increasing threat (5, 6). In a recent survey, *I. scapularis* ticks collected from the northwest quadrant of Wisconsin from 2011 to 2012 demonstrated a POWV infection frequency of 4.67% (7). This is similar in frequency to a survey conducted in that same area in 1998 (8). Although POWV is rarely diagnosed as a cause of encephalitis, infections can be severe, and neurologic sequelae are common (9). Additionally, the potential for concurrent transmission with other tick-borne pathogens, particularly *Borrelia burgdorferi*, the causative agent of Lyme disease, has not been previously studied in North America.

Similarly to other arboviral infections, POWV diagnosis is complex, requiring review of clinical and travel history in addition to knowledge of and access to diagnostic testing (10). Serologic testing remains the primary method for diagnosis of POWV infection, with an emphasis on the detection of POWV-specific IgM antibodies in serum or plasma. Until recently, commercial laboratory testing has been unavailable for POWV in the United States. Prior to this, a positive POWV IgM enzyme immunoassay (EIA) result confirmed by plaque reduction neutralization test (PRNT), a 4-fold or greater increase in titers between acute- and convalescent-phase sera, or culture or direct identification of virus-specific nucleic acids at a state public health laboratory or the Centers for Disease Control and Prevention (CDC) (11) has been the mainstay of diagnostic testing.

We describe here a laboratory-developed, serologic test panel, commercially available at a reference laboratory, for the detection of IgG and IgM antibodies to POWV in serum and plasma samples. The first test in the panel is a highly sensitive, commercial TBE-C screen by EIA. Per the manufacturer, cross-reactivity with other flaviviruses is expected, particularly with West Nile virus (WNV) and dengue virus (DENV) antibody-positive samples. Samples that are screen positive are then confirmed for POWV by indirect immunofluorescence assay (IFA). Performance characteristics of the test panel were optimized, and validation studies were performed to assess the analytical sensitivity, reproducibility, and specificity/cross-reactivity of the serologic test panel for use in routine diagnostic testing.

## RESULTS

### Assay optimization and analytical sensitivity.

**FIG 1:**
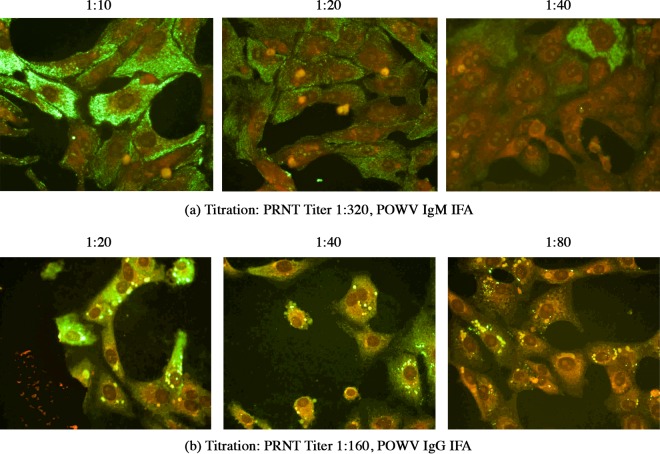
Titration of acute-phase tick-borne disease (TBD) samples in indirect immunofluorescence assay (IFA) to determine optimal screening dilutions. (a) Serial 2-fold dilutions of acute-phase TBD sample with Powassan virus (POWV) plaque reduction neutralization test (PRNT) titer of 1:320 to determine optimal screening dilution for IgM IFA. (b) Serial 2-fold dilutions of acute-phase TBD sample with POWV PRNT titer of 1:160 to determine optimal screening dilution for IgG IFA.

No difference in sensitivity was observed between the viral strains tested or between lots of slides. A sample dilution of 1:20 for IgM and 1:40 for POWV IgG IFAs demonstrated the optimal balance between sensitivity and nonspecific background staining (Fig. 1). Tick-borne disease (TBD) samples with titers of 1:320 and 1:160 in the plaque reduction neutralization test using a 90% reduction cutoff (PRNT_90_) were assayed at optimized screening dilutions to confirm. All but one of the POWV encephalitis samples obtained from the New York State Department of Health (NYSDOH) with PRNT_90_ titers of 1:20 were detected by the TBE-C EIA screen and confirmatory POWV IFA. A PRNT_90_ titer of 1:20 was determined to be the limit of detection (LOD) for the serologic test panel and was confirmed as such using known PRNT_90_-positive samples (Table 1). At these screening dilutions, the serologic panel showed an analytical sensitivity of 89% (Table 1). Reproducibility studies showed 100% accordance (*k* = 1.0).

**TABLE 1:** Summary of POWV serologic data for arbovirus encephalitis panel sample set^a^

Sample no.	Result for assay:				
	TBE-C EIA IgM	POWV IFA IgM	TBE-C EIA IgG	POWV IFA IgG	POWV PRNT_90_ titer
1	+	ND^b^	ND	≥1:40	1:5,120
2	+	ND	+	≥1:40	1:640
3	+	ND	+	≥1:40	1:2,560
4	ND	≥1:20	+	≥1:40	1:320
5	+	≥1:20	ND	≥1:40	1:5,120
6	ND	≥1:20	+	≥1:100	1:10,240
7	+	ND	+	≥1:100	1:40,960
8	ND	ND	ND	ND	1:20
9	+	≥1:20	ND	≥1:40	1:20

a Overall POWV results were as follows: *n* = 9 (8 positive, 1 negative) and overall test panel sensitivity of 89%.

b ND, not detected at screening dilutions of 1:101 for TBE-C EIA, 1:20 for POWV IgM IFA, and 1:40 for POWV IgG IFA.

### Analytical specificity.

**FIG 2:**
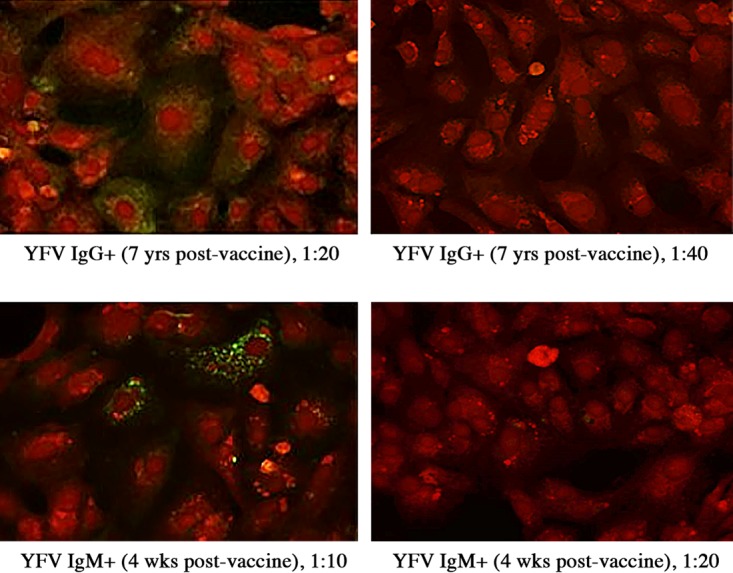
Yellow fever virus (YFV) vaccine recipient plasma samples in Powassan virus (POWV) indirect immunofluorescence assay (IFA) to determine optimal screening dilutions to eliminate cross-reactivity. (Top) YFV IgG-positive sample 7 years postvaccine assayed at 1:20 (left) and 1:40 (right) dilutions in IgG IFA. (Bottom) YFV IgM-positive sample 4 weeks postvaccine assayed at 1:10 (left) and 1:20 (right) dilutions in POWV IgM IFA.

Due to limited sample volume availability for the heterologous-flavivirus (HF) sample set, only yellow fever virus (YFV) vaccinee samples could be analyzed in replicate runs at two different dilutions to check IFA specificity. A sample known to be positive for YFV IgG antibodies (vaccinee 3) had a positive POWV IgG IFA result at a 1:20 dilution but was negative at a 1:40 dilution. A sample known to be positive for YFV IgM antibodies (vaccinee 1) had a positive POWV IgM IFA result at a 1:10 dilution but was negative at a 1:20 dilution (Fig. 2). Both of these samples were negative when tested at the optimized screening dilutions used for the panel. Only one recent YFV vaccine sample demonstrated cross-reactivity in the POWV IgG IFAs (Table 2) when assayed at optimal screening dilutions.

**TABLE 2:** Summary of data for HF sample set included in POWV IgG IFA analytical specificity studies^*a,c,d*^

Cluster and sample identifier (source)	Phylogenetic clade	Antigenic complex	Result for assay:	
			TBE-C EIA IgG	POWV IFA IgG
Tick-borne virus cluster^b^				
TBEV IgG (Euroimmun)	IV	TBE	+	≥1:40
TBEV IgG/M^+^ patient serum	IV	TBE	+	≥1:40
Mosquito-borne virus cluster				
DENV IgG (Euroimmun)	IX	DEN	+	ND
DENV IgG (SeraCare)	IX	DEN	+	≥1:40
DENV IgM (SeraCare)	IX	DEN	+	≥1:40
WNV IgG 1 (SeraCare)	XIV	JE	+	≥1:40
WNV IgG 2 (SeraCare)	XIV	JE	+	≥1:40
WNV IgG (Euroimmun)	XIV	JE	+	ND
WNV IgM 1 (SeraCare)	XIV	JE	+	ND
WNV IgM 2 (SeraCare)	XIV	JE	+	≥1:40
JEV IgG (Euroimmun)	XIV	JE	+	ND
YFV IgG (Euroimmun)	VII	Not classified	+	ND
YFV vaccinee serum 1	VII	Not classified	ND	ND
YFV vaccinee serum 2	VII	Not classified	+	ND
YFV vaccinee serum 3	VII	Not classified	+	ND
YFV vaccinee serum 4	VII	Not classified	ND	ND
YFV vaccinee serum 5	VII	Not classified	ND	ND
YFV vaccinee serum 6	VII	Not classified	ND	ND
YFV vaccinee serum 7	VII	Not classified	ND	≥1:40

a Phylogenetic and antigenic classification based on the work of Kuno et al. (24).

b Eighty-six percent positive at protein level for members of tick-borne virus cluster.

c Abbreviations: DENV, dengue virus; WNV, West Nile virus; TBEV, tick-borne encephalitis virus; JEV, Japanese encephalitis virus; YFV, yellow fever virus; ND, not detected at screening dilutions of 1:101 for TBE-C EIA and 1:40 for POWV IgG IFA.

d IFA results for POWV-negative samples (*n* = 19) were as follows: 2 from the tick cluster, both positive; 17 from the mosquito cluster, 6 positive and 11 negative; overall IgG IFA positive specificity, 65%. The analytical specificity calculation includes the mosquito-borne virus cluster only (*n* = 17).

The HF samples demonstrated significant cross-reactivity in the TBE-C IgG EIA, but at a 1:40 dilution, the POWV IgG IFA specificity for non-TBE-C HF samples was 65% (Table 2). This is comparable to other commercial IFAs. Fewer HF samples demonstrated cross-reactivity in the TBE-C IgM EIA than in the TBE-C IgG EIA, and no cross-reactivity was seen in the POWV IgM IFA with any of the HF samples run at screening dilutions (Table 3).

**TABLE 3:** Summary of data for HF sample set included in POWV IgM IFA analytical specificity studies^*a,c,d*^

Cluster and sample identifier (source)	Phylogenetic clade	Antigenic complex	Result for assay:	
			TBE-C EIA IgM	POWV IFA IgM
Tick-borne virus cluster^b^				
TBEV IgM (Euroimmun)	IV	TBE	+	ND
TBEV IgG/M^+^ patient serum	IV	TBE	+	ND
Mosquito-borne virus cluster				
DENV IgM (Euroimmun)	IX	DEN	ND	ND
DENV IgM (SeraCare)	IX	DEN	ND	ND
DENV IgG (SeraCare)	IX	DEN	ND	ND
WNV IgM 1 (SeraCare)	XIV	JE	ND	ND
WNV IgM 2 (SeraCare)	XIV	JE	ND	ND
WNV IgM (Euroimmun)	XIV	JE	ND	ND
WNV IgG 1 (SeraCare)	XIV	JE	ND	ND
WNV IgG 2 (SeraCare)	XIV	JE	ND	ND
JEV IgG (Euroimmun)	XIV	JE	+	ND
YFV vaccinee serum 1	VII	Not classified	+	ND
YFV vaccinee serum 2	VII	Not classified	+	ND
YFV vaccinee serum 3	VII	Not classified	ND	ND
YFV vaccinee serum 4	VII	Not classified	ND	ND
YFV vaccinee serum 5	VII	Not classified	ND	ND
YFV vaccinee serum 6	VII	Not classified	ND	ND
YFV vaccinee serum 7	VII	Not classified	+	ND

a Phylogenetic and antigenic classification based on the work of Kuno et al. (24).

b Eighty-six percent positive at protein level for members of tick-borne virus cluster.

c Abbreviations: DENV, dengue virus; WNV, West Nile virus; TBEV, tick-borne encephalitis virus; JEV, Japanese encephalitis virus; YFV, yellow fever virus; ND, not detected at screening dilutions of 1:101 for TBE-C EIA and 1:20 for POWV IgM IFA.

d IFA results for POWV-negative samples were as follows: *n* = 18 (2 from tick cluster and 16 from mosquito cluster), 0 positive samples, and POWV IgM IFA positive specificity of 100% (analytical specificity calculation includes mosquito-borne virus cluster only [*n* = 16]).

### Clinical testing.

Twenty-two (20%) of the TBD samples had TBE-C optical density (OD) ratios above baseline and were tested in the POWV confirmatory IFA. One-half of these samples (*n* = 11, Table 4) were IFA positive for antibodies to POWV. Two of the three POWV IgG IFA-positive samples were PRNT_90_ positive (samples 13 and 19). The third sample (sample 12) was negative in the PRNT_90_ and West Nile virus (WNV) IgG positive, demonstrating evidence of cross-reactivity in the POWV IgG IFA. All eight samples that were positive only in the POWV IgM IFA were negative in the PRNT. POWV nucleic acid was detectable by reverse transcription-PCR (RT-PCR) in one sample (sample 16 in Table 4). POWV seroprevalence for the TBD sample set was determined to be 9.4% (10 of 106).

**TABLE 4:** Summary of POWV serologic data for TBD sample set

Sample no.	TBE-C EIA IgM	POWV IFA IgM	TBE-C EIA IgG	POWV IFA IgG	POWV PRNT_90_ titer
10	+	≥1:20	ND^a^	ND	ND
11	+	≥1:20	ND	ND	ND
12^b^	ND	ND	+	≥1:40	ND
13	ND	ND	+	≥1:40	1:160
14	+	≥1:20	ND	ND	ND
15	+	≥1:20	ND	ND	ND
16^c^	+	≥1:20	ND	ND	ND
17	+	≥1:20	ND	ND	ND
18	+	≥1:20	ND	ND	ND
19	+	≥1:20	+	≥1:40	1:320
20	+	≥1:20	ND	ND	ND

a ND, not detected at screening dilutions of 1:101 for TBE-C EIA, 1:20 for POWV IgM IFA, and 1:40 for POWV IgG IFA and 1:10 for POWV PRNT.

b Sample tested positive for IgG antibodies to WNV.

c POWV RNA detected in RT-PCR test.

Of the 100 non-TBD samples from areas where Lyme disease is endemic, 23% (*n* = 23) had TBE-C EIA OD ratios higher than baseline and were tested by POWV IFA. Six of the 23 EIA-positive samples were IFA positive for antibodies to POWV (Table 5), with four samples being IgG IFA positive and two samples IFA positive for IgG and IgM. Three of the four IgG IFA-positive samples were positive for WNV IgG, again demonstrating cross-reactivity of the IgG with WNV. The fourth sample was negative by PRNT_90_. POWV seroprevalence for the non-TBD sample set was determined to be 2% (2 of 100). Patients with suspected TBD, who were tested for Lyme disease, are significantly (*P* = 0.034) more likely to have evidence of POWV infection than asymptomatic patients from an area where Lyme disease is endemic but who did not have a history of recent tick exposure.

**TABLE 5:** Summary of serologic data available for TBE-C EIA-positive non-TBD samples from areas where Lyme disease is endemic

Sample no.	TBE-C EIA IgM	POWV IFA IgM	WNV EIA IgM	TBE-C EIA IgG	POWV IFA IgG	WNV EIA IgG
21	ND^a^	ND	ND	+	ND	ND
22	+	ND	ND	+	≥1:40	+^b^
23	ND	ND	ND	+	ND	+
24	ND	ND	ND	+	ND	ND
25	ND	ND	ND	+	≥1:40	+
26	+	ND	ND	ND	ND	ND
27	ND	ND	ND	+	≥1:40	+^b^
28	+	ND	ND	ND	ND	ND
29	ND	ND	ND	+	ND	ND
30	+	≥1:20	ND	+	≥1:40	+
31	+	ND	ND	ND	ND	ND
32	+	ND	ND	ND	ND	ND
33	ND	ND	ND	+	ND	+
34	+	ND	ND	+	ND	ND
35	+	ND	ND	ND	ND	ND
36	ND	ND	ND	+	ND	ND
37^c^	+	ND	ND	ND	≥1:40	ND
38	+	ND	ND	ND	ND	ND
39^c^	+	≥1:20	ND	+	≥1:40	ND
40	+	ND	ND	ND	ND	ND
41	ND	ND	ND	+	ND	+
42	ND	ND	ND	+	ND	ND
43	+	ND	ND	ND	ND	ND

a ND, not detected at screening dilutions of 1:101 for TBE-C and WNV EIA, 1:20 for POWV IgM IFA, and 1:40 for POWV IgG IFA.

b Sample tested positive for IgG antibodies to WNV with an OD ratio of >3.0.

c Sample was tested in POWV PRNT_90_ and not detected.

Six of the 22 control samples from an area in which Lyme disease was not endemic had TBE-C EIA OD ratios above the cutoff in the IgM assay, but none were positive in the POWV IFAs (0 of 22). None of these samples were above the cutoff in the TBE-C IgG EIA.

## DISCUSSION

Studies confirm that serologic cross-reactions among the flaviviruses are significant and more frequently observed with IgG antibody detection assays. Both commercial EIAs and IgG IFAs demonstrate poor specificity overall, with IFA being slightly better than EIA. The overall specificity of flavivirus IgG EIAs is reported between 16 and 38% and improves slightly to 29 to 84% for IgG IFAs (12). The addition of the POWV IgG IFA to the TBE-C EIA screen eliminated 35% of the cross-reactivity seen with the EIA alone. The immunological maturation of the IgG antibody response tends to foster the formation of antibodies directed to epitopes with greater likelihood for cross-reactivity (13). While the specificity of flavivirus PRNTs detecting neutralizing IgG antibodies is high, this often comes at the cost of assay sensitivity. A PRNT_50_ tends to be more sensitive in detecting early antibody responses, given the less stringent endpoint of >50% reduction in plaques, versus the highly specific >90% plaque reduction described here (14). Also, low correlation between PRNT and IFA titer is not surprising since the IFA detects antibodies to a variety of structural and nonstructural viral proteins, whereas the PRNT primarily detects neutralizing antibodies directed to the envelope (E) protein of the virus (13). When tested against non-tick-borne flavivirus samples, the POWV IgG IFA described here demonstrated an analytical specificity of 65%, comparable to that of other commercial flavivirus IgG IFAs (15).

The overall cross-reactivity associated with detection of flavivirus IgM antibodies is significantly lower than that seen with IgG antibodies, and IgM IFA specificity was 20 to 30% higher than IgM EIA when patients with suspected flavivirus infections were screened for several different viruses (12). The addition of the POWV IgM IFA to the TBE-C EIA screen eliminated 55% of the cross-reactivity seen with the IgM EIA alone. Many commercially available flavivirus IgM-specific serologic tests report sensitivity lower than that seen with IgG assays, due primarily to the fact that PRNT is considered the gold standard for the differential serodiagnosis. Published reports confirm that, in patients with primary flavivirus infection, the IgM response likely consists of antibodies with minimal to no virus-neutralizing capacity (16, 17). In a study assessing the performance of a commercially available WNV IgM EIA, 14 of 44 CDC IgM protocol-negative samples were found positive. The apparent lack of IgM specificity observed with that assay may be attributed to the use of PRNT to confirm IgM positives as part of the CDC protocol (18). In studies where convalescent-phase samples can be collected from patients 2 to 4 weeks after infection, the use of PRNT may be helpful to confirm the accuracy of acute-phase detection of IgM. In the studies presented here, we were limited by design to samples collected only at the time of clinical presentation, and therefore, follow-up convalescent-phase samples were not available. The lack of a reliable and accurate predicate assay for use as a gold standard when developing flavivirus IgM assays requires further study.

According to the World Health Organization (WHO), a single IgM-positive result for arboviral diagnosis is considered a presumptive positive and should be confirmed by RT-PCR, virus isolation, and/or PRNT to discriminate potentially cross-reactive antibodies (19). In the current study, one POWV IgM IFA-positive sample was confirmed positive by POWV RT-PCR with the remaining IgM IFA-positive samples lacking detectable viral genome. Studies performed with WNV-positive patients show that viremia can be detected for an average of 7 days and that IgM antibody becomes detectable a median of 4 days after the midpoint of the interval for RNA detection, for an overlap of only 1 to 2 days (20). Therefore, RT-PCR cannot function as a sensitive confirmatory assay for IgM-positive samples across all time points.

The current state of diagnostics for POWV infection includes initial serological testing performed using IgM capture enzyme-linked immunosorbent assay (ELISA) and IgG ELISA with confirmatory testing utilizing PRNT, a 4-fold or greater increase in antibody titer between acute- and convalescent-phase sera, virus isolation, or detection of virus-specific RNA. Only a few state health labs and the CDC offer this testing, and the labor-intensive nature of the confirmatory tests often delays the reporting of final results beyond the period of clinical utility. In addition, testing is usually limited to patients presenting with neurologic complications; therefore, little is known regarding the overall prevalence of and clinical symptoms associated with POWV infection. Limited data regarding cocirculation of Lyme disease and POWV seroprevalence in the Midwest have been published (21). The test panel described here allows for the simple and efficient detection of acute POWV infection. However, due to the cross-reactivity seen for all flavivirus IgG assays, it is imperative that exposure to other flaviviruses and travel and vaccination history be considered when interpreting IgG test results. This panel is a diagnostic tool that can play an important role in further defining the full spectrum of POWV disease.

## MATERIALS AND METHODS

### Samples for assay development. (i) Serum samples submitted for arbovirus encephalitis panel testing.

Optimization and analytical sensitivity studies were performed using POWV antibody-positive serum samples confirmed by PRNT using a 90% reduction cutoff (PRNT_90_) (22). These samples had been submitted to the New York State Department of Health (NYSDOH) Arbovirus Laboratory (*n* = 9) for testing against an arbovirus encephalitis panel.

### (ii) Heterologous-flavivirus sample set.

The heterologous-flavivirus (HF) sample set consists of 11 human serum/plasma samples known to be IgM antibody positive and 12 samples known to be IgG antibody positive to heterologous flaviviruses, including dengue virus (DENV) and West Nile virus (WNV) (SeraCare’s bank of disease state biological materials; Milford, MA) and Japanese encephalitis virus (JEV), yellow fever virus (YFV), and TBEV (Euroimmun US). Another seven samples collected from YFV strain 17D vaccine recipients at various time points postvaccination were included in the HF sample set.

### Clinical samples to assess seroprevalence. (i) TBD sample set.

The tick-borne disease sample set consists of 106 serum/plasma samples submitted for Lyme disease testing from patients residing in Wisconsin, an area in which Lyme disease is endemic (23). Patients were considered to have suspected TBD if a Lyme disease serology test was ordered by their provider. Eleven serum samples were submitted to Coppe Laboratories for routine Lyme disease testing, deidentified, and included in this study. Ninety-five serum/plasma samples provided by the Marshfield Clinic Research Foundation (MCRF) were residual diagnostic samples which had been submitted to Marshfield Clinic for Lyme disease testing. The 95 samples from MCRF were part of an institutional review board (IRB)-approved research protocol.

### (ii) Non-TBD sample set.

The non-TBD sample set collected from areas in which Lyme disease is endemic consists of 100 serum/plasma samples collected from patients without symptoms of TBD and without history or evidence of recent tick exposure. Fifty heparinized whole-blood samples from healthy, asymptomatic adults were purchased from Analytical Biological Services (ABS), Inc. (Wilmington, DE), and processed in-house to plasma. These samples were collected locally for biospecimen banking, in the northeastern region of the United States, where Lyme disease is endemic (23). Fifty plasma samples were collected by the Marshfield Clinic from residual diagnostic samples submitted for routine cholesterol or complete metabolic panel screening. These samples were collected from subjects living in the same geographic area as the TBD subjects and were part of the same IRB-approved protocol.

### (iii) Control sample set.

The control sample set from a region where Lyme disease is not endemic consists of 22 samples collected from patients residing near Reno, NV. These samples, submitted to Coppe Laboratories for human herpesvirus testing, were deidentified and included in the study.

### Test methods. (i) TBE-C EIA method.

Samples were screened for the presence of IgG and IgM antibodies to TBE-C using anti-TBEV ELISA IgG (Euroimmun catalog no. EI-2661-9601G) and anti-TBEV ELISA IgM (Euroimmun catalog no. EI-2661-9601M) per the manufacturer’s recommended assay protocol. These assays employ inactivated TBEV antigens of strain K23. Optical density (OD) ratio values were calculated for each sample in reference to a calibrator provided with the test kit. Any samples with an OD ratio that exceeded the baseline value were reflexed to confirmatory POWV IFA.

### (ii) IFA method.

An in-house strain of Powassan virus lineage II, sequence-confirmed deer tick virus (DTV) isolated from an *I. scapularis* tick pool, along with viral reference strains Powassan LB and DTV CT390 (World Reference Center for Emerging Viruses and Arboviruses, Galveston, TX), was cultured in Vero cells and used to prepare IFA substrate slides. Three different culture techniques were tested to determine the best slide manufacturing conditions. Vero cells infected with POWV were grown on eight-well chamber slides using optimized culture conditions. Slides were fixed in 2% neutral buffered formalin (NBF) followed by 100% cold methanol and stored at −20°C until use. Prior to testing, substrate slides were warmed for 30 min in a humidity chamber at room temperature and blocked with 2% normal goat serum (Vector Laboratories, Burlingame, CA) for an additional 30 min. Patient serum and/or plasma samples, positive and negative controls, were diluted 1:40 in 2% normal goat serum for IgG analysis and 1:20 in Eurosorb (Euroimmun US) anti-human IgG antibody buffer for IgM analysis, added to slide wells, and incubated for 60 min at 37°C. After 1 h of incubation, the slides were washed 3 times with phosphate-buffered saline (PBS), and 50 μl of DyLight 488 anti-human IgG (Vector Laboratories) diluted 1:300 or 50 μl of fluorescein anti-human IgM (mu-specific) secondary antibody (Vector Laboratories) diluted 1:200 was added to each well. The slides were incubated for 30 min and washed three times in PBS. The slides were counterstained with Evans blue, rinsed with distilled water, mounted using Vectashield mounting medium (Vector Laboratories), and examined using an epifluorescence microscope. Positive- and negative-control samples were included in all IFA runs.

### (iii) Optimization of IFA.

Titrations of fluorochrome-conjugated anti-human secondary antibodies were performed to determine the optimal concentration for the assay. Sample titration experiments were performed to determine optimal screening dilutions for IFAs that yield acceptable sensitivity while minimizing cross-reaction with the heterologous-flavivirus (HF) sample set. Low-PRNT_90_-titer samples were analyzed in duplicate assays at optimal screening dilutions to determine the lower limit of detection (LOD) for the IFA. Reproducibility was assessed by testing 26 samples (17 positive samples and 9 negative samples) in duplicate assays.

### (iv) WNV method.

Samples were tested for the presence of IgG and IgM antibodies to WNV by qualitative EIA (Euroimmun US), and results were analyzed per the manufacturer’s recommended protocol.

### (v) Clinical testing.

All 106 TBD samples were tested for TBE-C by EIA. Any samples with TBE-C OD ratios above baseline were tested with the optimized POWV IFA and WNV EIA. POWV IFA-positive samples were submitted to NYSDOH for PRNT. A PRNT_90_ rather than a PRNT_50_ was used to ensure specificity, though the expected sensitivity is lower (22).

All 100 non-TBD samples were tested for TBE-C and WNV by EIA. Samples with higher-than-baseline TBE-C OD ratios were tested with the optimized POWV IFA. The two Marshfield Clinic samples that were POWV IFA positive were also tested by PRNT_90_.

The 22 control samples were tested for TBE-C by EIA, and any samples with above-baseline OD ratios reflexed to confirmatory IFA.

### (vi) Statistical analysis.

Analytical sensitivity was calculated as (true positives)/(true positives + false negatives) × 100. Analytical sensitivity was determined using the arbovirus encephalitis panel. Analytical specificity was calculated as (true negatives)/(true negatives + false positives) × 100. The limit of detection was calculated as the lowest PRNT_90_ titer detected in the POWV IgG assay. Analytical specificity was determined using the HF sample set. Seroprevalence was calculated for each of the TBD, non-TBD, and control sample sets. Due to the inability of PRNT_90_ to be used for confirmation of POWV IgM and the lack of cross-reactivity seen with the HF samples, samples that were POWV IgM IFA positive or POWV IgG positive and confirmed by PRNT_90_ were presumed to be true positives. For samples that were negative for POWV IgM by IFA, POWV IgG was considered a false positive if WNV IgG EIA was positive or PRNT_90_ was negative.
